# Identification of a two-gene prognostic model associated with cytolytic activity for colon cancer

**DOI:** 10.1186/s12935-021-01782-6

**Published:** 2021-02-08

**Authors:** Xiaoye Jiang, Zhongxiang Jiang, Lichun Xiang, Xuenuo Chen, Jiao Wu, Zheng Jiang

**Affiliations:** Departments of Gastroenterology, Chongqing Medical University First Affiliated Hospital, Chongqing, 400016 China

**Keywords:** Prognostic model, Immune profile, Colon cancer, Gtex, TCGA

## Abstract

**Background:**

Increasing evidence has shown that cytolytic activity (CYT) is a new immunotherapy biomarker that characterises the antitumour immune activity of cytotoxic T cells and macrophages. In this study, we established a prognostic model associated with CYT.

**Methods:**

A prognostic model based on CYT-related genes was developed. Furthermore, aberrant expression of genes of the model in colon cancer (CC) was identified by reverse transcription-quantitative polymerase chain reaction (RT-qPCR) and immunohistochemistry (IHC) assays. Next, the correlation between the model and T-cell infiltration in the CC microenvironment was analysed. The Tumour Immune Dysfunction and Exclusion (TIDE) algorithm and subclass mapping were used to predict clinical responses to immune checkpoint inhibitors.

**Results:**

In total, 280 of the 1418 genes were differentially expressed based on CYT. A prognostic model (including HOXC8 and MS4A2) was developed based on CYT-related genes. The model was validated using the testing set, the whole set and a Gene Expression Omnibus (GEO) cohort (GSE41258). Gene set enrichment analysis (GSEA) and other analyses showed that the levels of immune infiltration and antitumour immune activation in low-risk-score tumours were greater than those in high-risk-score tumours. CC patients with a low-risk-score showed more promise in the response to anti-immune checkpoint therapy.

**Conclusions:**

Overall, our model may precisely predict the overall survival of CC and reflect the strength of antitumour immune activity in the CC microenvironment. Furthermore, the model may be a predictive factor for the response to immunotherapy.

## Background

In the Global Cancer Statistics 2018 report, colon cancer (CC) accounted for approximately 1.1 million new patients and 551,269 deaths [1]. Currently, there are many established treatments for CC, including surgical resection, biological targeted therapy, and chemotherapy [2], but the mortality rate remains high. To improve the survival rate of patients, their prognosis should be closely monitored to adjust the treatment plan. Therefore, a powerful prognostic index is needed. In addition, due to the poor prognosis after standard treatment, immunotherapy is being explored as adjuvant therapy [3].

Programmed death 1 (PD1) is an important immune checkpoint receptor that regulates the function of T cells. Tumour cells can escape the immune surveillance of T cells through the PD1/PDL1 pathway to achieve the effect of immune escape. Anti-PD1/PDL1 immune checkpoint antibody treatment can enhance antitumour immunity [4]. In the past 5 years, although anti-PD1/PDL1 immune checkpoint antibodies have achieved great success in the treatment of colon cancer, they have not been effective for most patients [5]. Studies have shown that cytotoxic T cells, natural killer cells and other immune cell infiltration conditions can reflect the strength of antitumour immunity and are associated with the efficacy of immune checkpoint inhibitors [6]. In addition, they are closely associated with the clinical prognoses of many tumours, including colorectal cancer and squamous cell carcinoma of the larynx and pharynx [7, 8]. Michael S. et al. identified the cytolytic activity (CYT) score as a new immunotherapy biomarker that can characterise the antitumour immune activity of CD8 + cytotoxic T cells and macrophages [9]. Therefore, it is necessary to explore genes associated with the CYT level. Interestingly, patients with colon cancer and a high CYT may be more sensitive to anti-immune checkpoint therapy [10]. Hence, exploring gene markers associated with CYT is needed to predict the prognosis of CC.

For the first time, we used RNA sequencing data from The Cancer Genome Atlas (TCGA) to identify CYT-related differentially expressed genes (DEGs). Next, the biological effects of these DEGs were further analysed through functional enrichment analysis. Finally, a two-gene prognostic model of CC was constructed and verified. The model has potential application value in patient management and can be used as a significant prognostic indicator. Most importantly, our prognostic model may reflect the strength of the antitumour immune response in the CC microenvironment and predict immunotherapeutic benefit in CC.

## Methods

### Colon adenocarcinoma (COAD) datasets and data processing

Several data sets from a public database were employed in this research. To address the imbalance between the tumour and normal data in TCGA database, we downloaded TOIL GTEx and TCGA RNA-seq datasets (including 349 non-cancerous colon samples and 290 CC samples) and TCGA COAD RNA-seq datasets (including 41 non-cancerous colon samples and 471 CC samples) from the UCSC Xena project (http://xena.ucsc.edu/) [11]. In the Homo_sapiens.GRCh38.96.chr.gtf document, the gene symbols were interpreted. The clinical data were gathered from the TCGA repository. The mean value of the RNA expression was utilised if multiple values were found.

From the repository of the Gene Expression Omnibus (GEO), information on gene expression profile matrices in GSE41258 (including 54 non-cancerous samples and 186 CC samples) from platform GPL96 and GSE39582 (including 19 normal samples and 566 tumour samples) was obtained [12, 13]. The groups of data were log2 transformed.

### Analysis of DEGs

In the TCGA COAD RNA-seq fragments per kilobase of transcript per million mapped reads (FPKM) cohort and the GSE39582 cohort, we calculated the CYT score using the geometric mean of GZMA and PRF1 [9]. We identified DEGs between the low-CYT groups (the bottom 25% of samples) and high-CYT groups (the top 25% of samples) using the limma package in R software [14]. To select the intersecting genes, DEGs between non-tumour colon samples and CC samples were screened, and the cut-off values were set as a log_2_|fold change| (log_2_FC) > 1 and a false discovery rate (FDR) < 0.05. Using the clusterProfiler R package, we performed gene ontology (GO) and Kyoto Encyclopedia of Genes and Genomes (KEGG) pathway analyses to investigate the potential molecular mechanisms of the DEGs, and the significance criteria were set to enrichment > 2 and P < 0.05 [15].

### Establishment of the CYT-related prognostic model

For survival analysis, 362 representative patients were included from the TCGA repository. They exhibited a follow-up duration greater than one month, and all relevant information, such as age, sex, tumour stage and tumour-node-metastasis (TNM) stage, was available for these patients. Tumour staging was classified using the American Joint Committee on Cancer (AJCC) staging system [16]. Prognostic genes were identified using univariate Cox regression analysis. Thereafter, the patients were divided into two cohorts (training cohort and testing cohort) randomly and equally. Additional file 1: Table S1 describes the characteristics of each set. In both sets, we ensured that the clinical parameters did not vary extensively. Moreover, the prognostic genes were selected through least absolute shrinkage and selection operator (LASSO)-penalised Cox regression analysis [17]. With the LASSO Cox regression model coefficients (β), we obtained a linear combination of the regression coefficient and then constructed the prognostic model. The risk score was calculated by using the following formula: (β_mRNA1_*mRNA1′s expression level) + (β_mRNA2_*mRNA2′s expression level) + ⋯ + (β_mRNAn_*mRNAn’s expression level). The R package survival and survminer were applied to determine the optimum cut-off evaluation [18]. The samples were sorted into either a high-risk or a low-risk cohort according to the cut-off. Then, a receiver operating characteristic (ROC) curve was generated by the R package survivalROC [19]. The Kaplan–Meier survival curve of the risk scores was generated using the R package survival. We then examined the estimated value of the model in the testing set, whole set and GSE41258 cohort.

### Reverse transcription-quantitative polymerase chain reaction (RT-qPCR)

We gathered 30 representative fresh CC samples and adjacent normal colon samples from the operating room of the Affiliated Hospital of Chongqing Medical University (Chongqing, China; October 2018 to September 2019). The resected tissues were snap-frozen in liquid nitrogen and stored at − 80 °C for RT-qPCR. The total RNA of the CC tissues and adjacent normal colon tissues was extracted using TRIzol reagent (Takara Biotechnology Co., Ltd., Dalian, China). We used a reverse transcription kit (Takara Biotechnology Co., Ltd.) to prepare cDNA and perform quantitative real-time PCR using TB Green™ Premix Ex Taq (Takara Biotechnology Co., Ltd.) and the Applied Biosystems StepOnePlus Real-Time PCR system. The mRNA expression was normalised to that of β-actin mRNA, and we applied the $2^{{\Delta {\text{C}}_{{\text{t}}} }}$ method to evaluate the relative expression levels of HOXC8 and MS4A2 [20]. All the primers were purchased from Takara (Dalian, China). The primers, NCBI reference sequences (HOXC8, MS4A2, and β-actin) and PCR parameters are presented in Additional file 2: Table S2. Each sample and each qPCR run were repeated thrice.

### Immunohistochemistry (IHC)

Forty paraffin sections of normal and CC samples were collected from the Department of Pathology of the Affiliated Hospital of Chongqing Medical University (April 2017 to August 2019), and the protein levels were determined by IHC. In Additional file 3: Table S3, the patient information and clinical characteristics are summarized. The primary antibodies used were: anti-MS4A2 antibody (ab158422; 1:250; Abcam) and anti-HOXC8 antibody (ab86236; 1:100; Abcam). Using a standard 3,3′-diaminobenzidine (DAB) protocol, the labelled antigens were visualised after applying of biotin-labeled goat anti-rabbit IgG. Next, the slides were counterstained lightly with haematoxylin. As a negative control, staining without primary antibody was performed. Image capture was performed using a Leica microscope (× 200 and × 400 magnification; Leica Microsystems GmbH, Wetzlar, Germany). We estimated the immunoreactivities as described previously with the use of ImageJ and IHC profiler plug [21]. The immunoreactivity scores were assigned from 1 to 4 (1: negative, 2: low positive, 3: positive, 4: high positive).

The patient groups for qPCR and IHC had no overlap. No patient had undergone chemoradiotherapy or other biological therapies before surgery. Informed consent was provided by the concerned patients based on which this research was approved by the Ethics Committee of the Affiliated Hospital of Chongqing Medical University.

### The model plays an independent prognostic role

To determine whether the model was independent of age, sex, TNM stage, and tumour stage, we performed univariate and multivariate analyses using the Cox regression model procedure, employing a forwarding stepwise method and SPSS. The cut-off was set as* P* < 0.05.

### Construction and validation of a predictive nomogram

To predict cancer prognoses, a nomogram was utilised [22]. Age, T stage, N stage, M stage, and risk score were used to construct a nomogram to predict the 1-, 3-, and 5-year overall survival (OS) of CC patients. The concordance index (C-index) and calibrations were used to test the nomogram’s validity. Moreover, using a bootstrap method with 1000 resamples, the C-index was measured to assess the nomogram’s discrimination ability. The calibration curve of the nomogram was then plotted to monitor its prediction tendencies with respect to the observations.

### Single-sample gene set enrichment analysis (ssGSEA) and gene set enrichment analysis (GSEA)

Using the ssGSEA score, the enrichment levels of the 29 immune signatures from every CC trial were measured in the TCGA COAD dataset [23]. The average of the standardised values for CD8^+^ T, T helper 1 (Th1), Th2, T follicular helper (Tfh), regulatory T (Treg) cells, tumour-infiltrating lymphocytes (TILs), and co-stimulated T cells were used to define the T-cell infiltration score (TIS) [24]. We performed GSEA on the risk score in the TCGA datasets to investigate GO and KEGG pathways; in which “c5.all.v7.1.symbols.gmt” and “c2.cp.kegg.v7.0.symbols.gmt” were used as the reference gene sets [25].

### Prediction of immunotherapeutic response

As previously described, potential immune checkpoint blockade responses were predicted using the Tumour Immune Dysfunction and Exclusion (TIDE) algorithm and subclass mapping. The TIDE algorithm can predict the immune checkpoint blockade response by simulating two main mechanisms of tumour immune evasion [26]. Using the online web tool TIDE (http://tide.dfci.harvard.edu/), the TIDE prediction score of every CC trial was measured in the TCGA COAD dataset. We then compared the expression profile of low/high risk scores with another published dataset (containing 47 melanoma patients who responded to immunotherapies) using submap (https://cloud.genepattern.org/gp) [27].

### Statistical analysis

R software v3.6.0 (R Foundation for Statistical Computing, Vienna, Austria) and SPSS (version 25.0) were used for statistical analysis and generating figures. Differences in the clinical parameters between the training and testing cohorts were assessed by Pearson’s *χ*^2^ test (or Fisher’s exact test). The Wilcoxon rank-sum test was used to compare the expression of HOXC8 and MS4A2 in different groups. Unless otherwise indicated, the cut-off was set at a *P*-value < 0.05.

## Results

### Study workflow

The study was conducted as shown in Additional file 6: Fig. S1. First, we obtained CYT-related DEGs, and then a two-gene prognostic model was established and validated. Finally, we verified the correlation between the model and T-cell immune infiltration.

### Acquisition and functional annotation of differentially expressed CYT-related genes

**Fig. 1:**
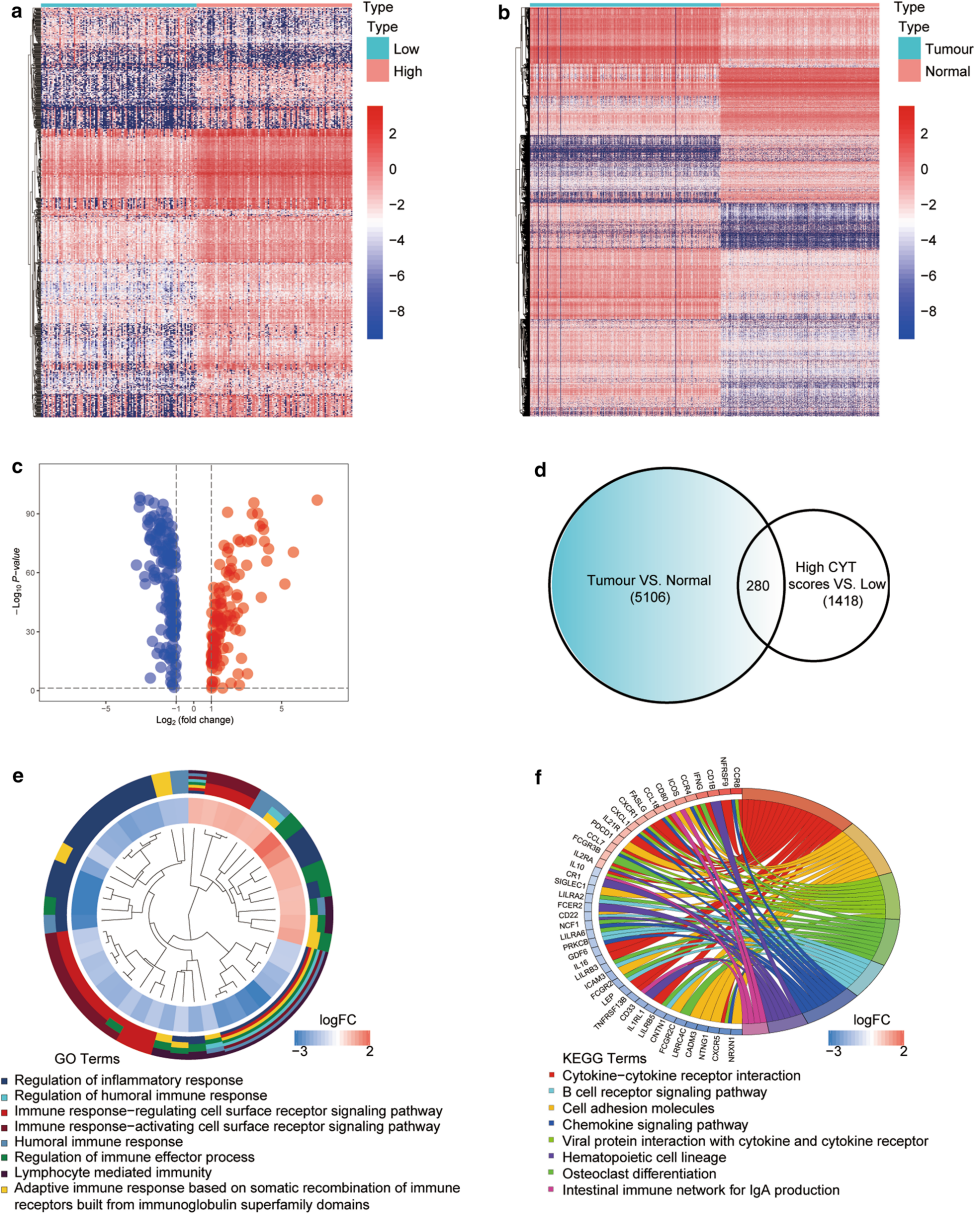
Differentially expressed genes (DEGs) related to cytolytic activity (CYT). **a** Heatmap of significantly DEGs based on CYT. **b** Heatmap demonstrating DEGs between CC and normal samples. Red indicates genes with a high expression level, and blue indicates genes with a low expression level. **c** Volcano plot of aberrantly expressed genes based on CYT scores and normal vs. tumour comparisons. **d** Venn diagram analysis of aberrantly expressed genes based on CYT score and normal vs. tumour comparison. **e** Gene ontology (GO) analysis of CYT‐related genes. **f** Kyoto Encyclopedia of Genes and Genomes (KEGG) analysis of CYT‐related genes

First, we obtained CYT-related genes (994 upregulated genes and 424 downregulated genes) by comparing high-/low-CYT samples (Fig. 1a). Subsequently, we identified 5106 DEGs (1710 upregulated genes and 3396 downregulated genes) by comparing normal and tumour samples (Fig. 1b). From the intersection of the two gene sets above, we extracted 280 differentially expressed CYT-related genes, including 160 downregulated genes and 120 upregulated genes (Fig. 1c, d). Furthermore, we conducted GO and KEGG enrichment analyses of the intersecting genes. GO showed enrichment of distinct biological processes, such as regulation of inflammatory response and regulation of immune effector process (Fig. 1e). These biological processes are all related to immune regulation. Cytokine-cytokine receptor interaction was the most enriched KEGG pathway of the genes (Fig. 1f). Thus, CYT-related genes are closely related to immune functions and pathways.

### Development of a two-gene-based prognostic model

**Fig. 2:**
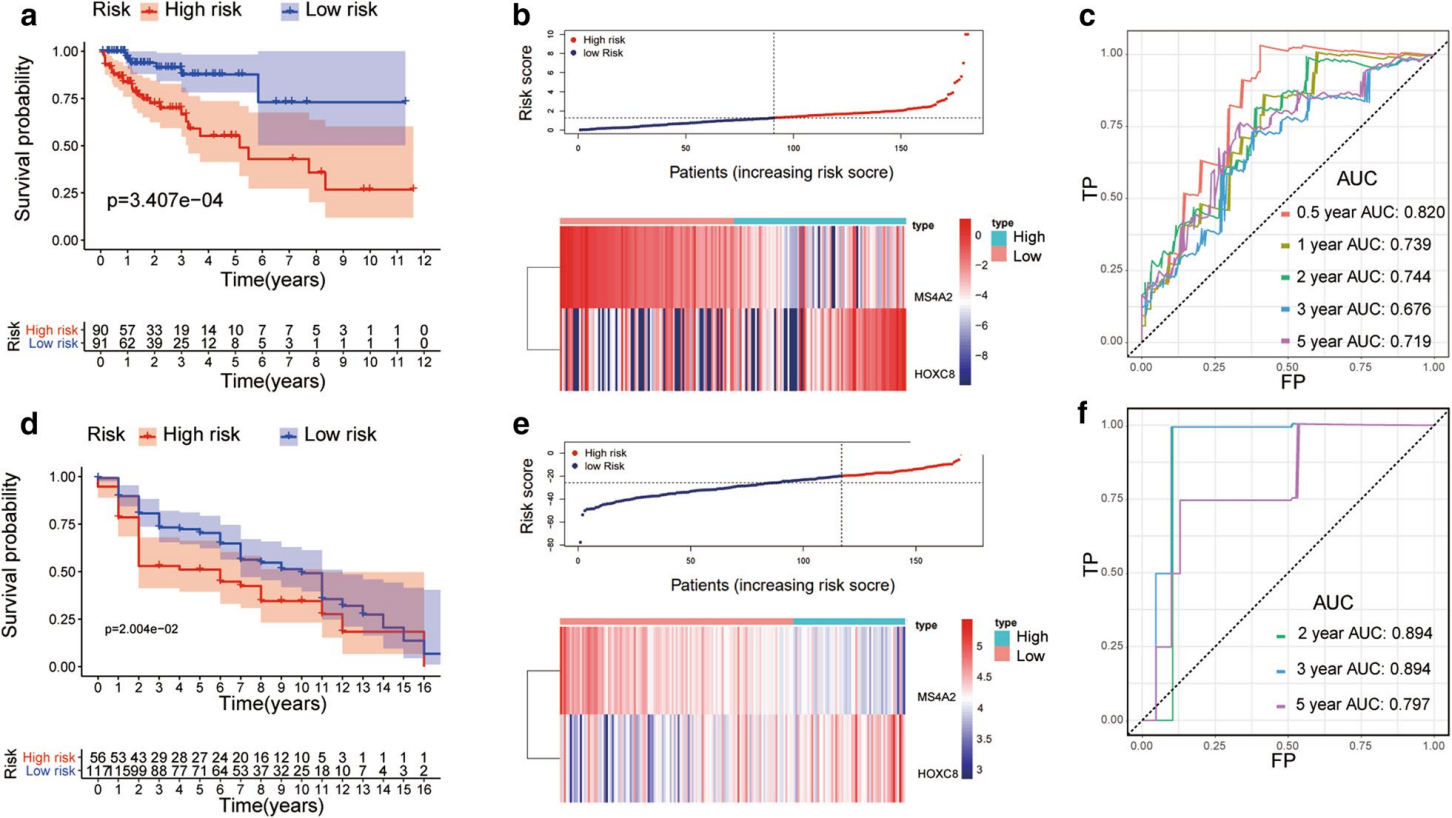
Kaplan–Meier survival, risk score and time-dependent receiver operating characteristic (ROC) curves of the CYT-related prognostic model for the training cohort **a**–**c** and testing cohort (GSE41258) **d**–**f** in CC. **a**, **d** Overall survival (OS) was significantly higher in the low-risk-score group than in the high-risk-score group. **b**, **e** The relationship between the risk score (upper panel) and expression of two prognostic immune genes (lower panel) is shown. **c**, **f** Time-dependent ROC curve analysis of the model

Using a univariate Cox regression model, we identified 26 genes significantly associated with OS (Additional file 4: Table S4) and removed the genes exhibiting the opposite tendency of expression and survival. Subsequently, we performed LASSO-penalised Cox analyses in the training cohort (n = 181) to further narrow the genes (Additional file 6: Fig. S2 a-b). Next, a CYT-related prognostic model was constructed using two genes: homeobox C8 (HOXC8) and membrane spanning 4-domains A2 (MS4A2). The risk score was calculated as follows: 1.52 * Expression_HOXC8_ + (− 2.81) * Expression_MS4A2_. The optimal cut-off point was -0.319. In the testing set and whole set, similar procedures were performed. Patients with a high risk score had a shorter OS than those with a low risk score in the training set [Hazard Ratio (HR) = 4.079; 95% confidence interval (CI) = 1.773–9.382; *P* < 0.001; Fig. 2a], testing set [HR = 2.16; 95% CI: 1.02–4.59; *P* < 0.05; Additional file 6: Fig. S3a], and the whole set (HR = 2.57; 95% CI: 1.215–4.369; *P* < 0.001; Additional file 6: Fig. S3d). In the training sets, the area under the curve (AUC) values for the 0.5-year, 1-year, 2-year, 3-year and 5-year OS were 0.820, 0.739, 0.744, 0.676 and 0.719, according to the ROC curves (Fig. 2c).

To ascertain the robustness of the model, its performance was tested using a GEO cohort (GSE41258). The results showed that patients within the high-risk group had a worse OS than those in the low-risk group (HR = 1.61; 95% CI: 1.03–2.52; Fig. 2d). The AUCs were 0.894, 0.894 and 0.797 at 2, 3 and 5 years, respectively (Fig. 2f). Together, the results demonstrate the decent performance of the model for survival prediction.

### External validation and experimental verification of the expression of the two genes in the model

**Fig. 3:**
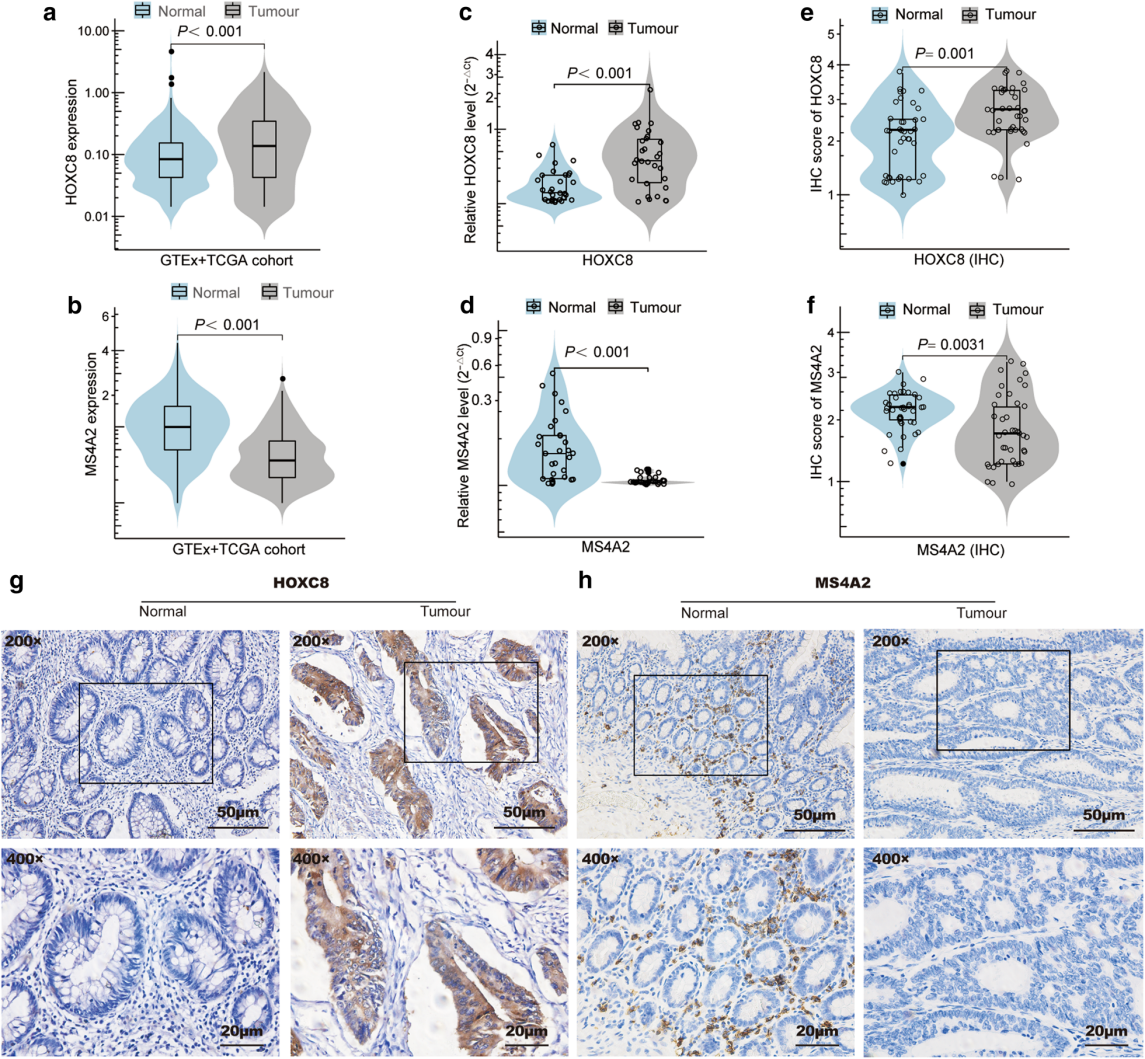
Expression of HOXC8 and MS4A2 in the TCGA + GTEx COAD cohort and in CC tissues. HOXC8 expression is elevated in CC tissues compared with control tissues; while MS4A2 expression is decreased in normal colon tissues compared with that in control tissues. **a**–**b** The HOXC8 and MS4A2 mRNA levels in colon clinical specimens and normal samples were analysed by using publicly available data sets (TCGA + GTEx COAD cohort). **c**–**d** The relative mRNA expression of HOXC8 and MS4A2 in 30 paired CC tumour and normal adjacent tissues was detected by RT-qPCR. **e**–**f** Statistical analysis of the immunoreactivity score of HOXC8 and MS4A2 between 40 pairs of CC tissues and corresponding non-tumour tissues. **g**–**h** Representative immunohistochemical images of HOXC8 and MS4A2 expression in clinical CC specimens and normal colon tissues (× 200, upper panel; × 400, lower panel)

To verify the expression of HOXC8 and MS4A2 in CC patients, we performed bioinformatics analysis by using the data from TCGA and GTEx datasets. The analysis indicated that the expression of HOXC8 mRNA was increased in 290 CC samples compared with 349 normal colon samples (Fig. 3a), while the expression of MS4A2 was decreased (Fig. 3b). The outcomes were verified by RT-qPCR. The mRNA levels of HOXC8 and MS4A2 in the 30 CC samples were significantly upregulated and downregulated, respectively, compared with those in the paired normal colon samples (Fig. 3c, d). The protein levels of HOXC8 and MS4A2 were examined by IHC. HOXC8 is localised in the cytoplasmic of tumour cells, and MS4A2 is expressed in interstitial cells. The outcomes indicated that HOXC8 protein expression was significantly upregulated in the CC samples compared with that in the normal samples (*P* = 0.001) (Fig. 3e, g). MS4A2 expression was significantly reduced in the CC samples (*P* = 0.0031) (Fig. 3f, h). The specific statistical parameters of the two genes in each group are shown in Additional file 5: Table S5.

### Relationship between the CYT-related prognostic model and clinicopathological features

**Fig. 4:**
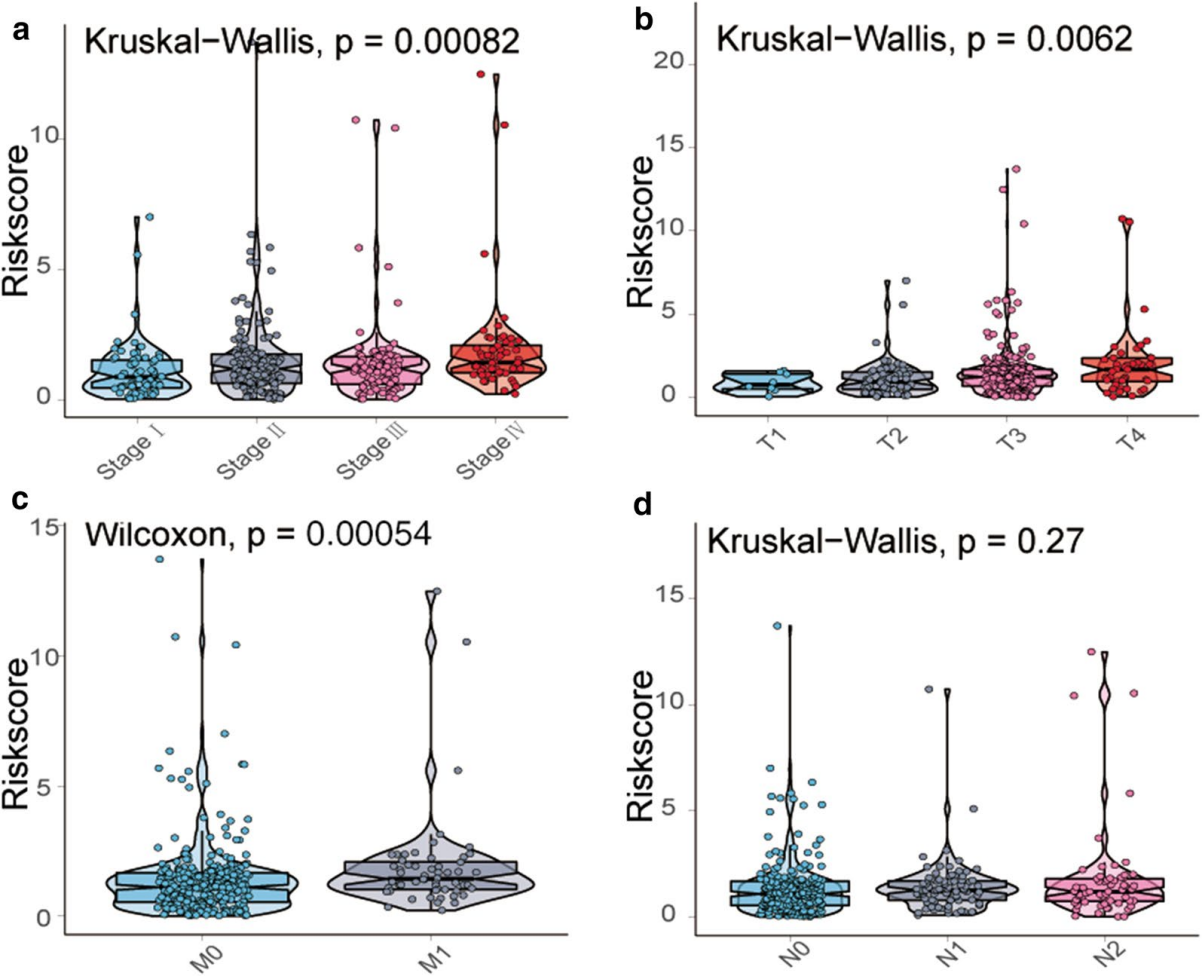
Relationship between the expression of the risk score and **a** TNM stage, **b** T stage, **c** M stage and **d** N stage in the TCGA cohort

To explore the relationships between the risk score and clinicopathological features, we used the TCGA cohort to analyse the distribution of the risk scores. We observed that the risk score was significantly positively associated with the tumour TNM stage (*P* < 0.001), T stage (*P* = 0.006) and M stage (*P* < 0.001). However, it was independent of the N stage (Fig. 4a–d). In addition, the results were verified using the IHC score (Additional file 6: Figure S3g–j).

**Fig. 5:**
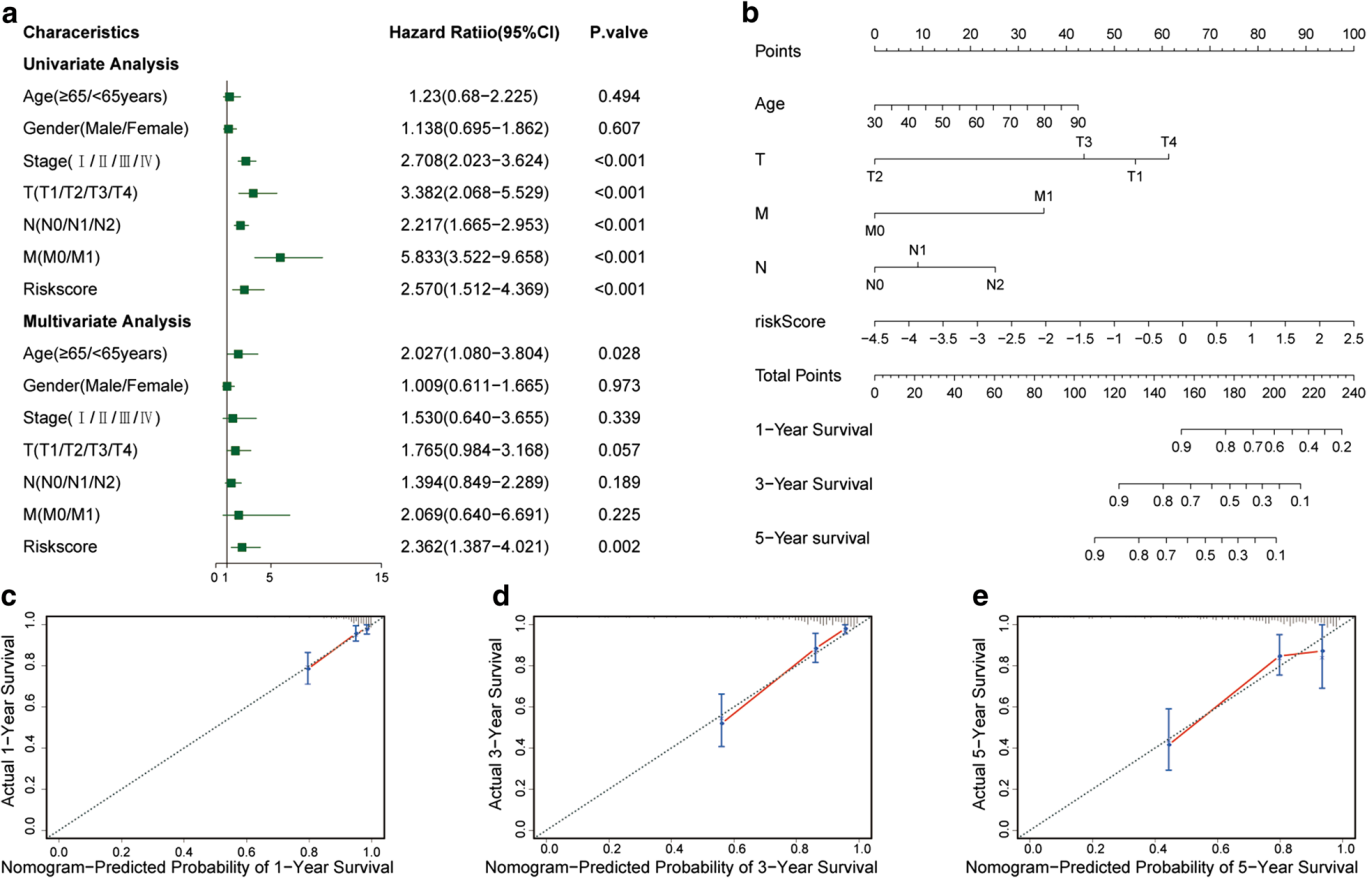
**a** Forest plot of univariate and multivariate Cox regression analyses in CC. Nomogram predicting the OS of CC patients. **b** For each CC patient, three lines are drawn upward to determine the points received from the three predictors in the nomogram. The sum of these points is located on the “Total Points” axis. Next, a line is drawn downward to determine the likelihood of the 1-, 3-, and 5-year OS. **c**–**e** Calibration plot of the nomogram for predicting the probability of OS at 1, 3 and 5 years

In univariate and multivariate Cox regression analyses, our model was associated with survival (*P* = 0.002). The median risk score of the model was the highest among other factors (HR = 2.362; 95%CI = 1.441–4.525) (Fig. 5a). These findings supported that the model was an independent prognostic factor.

To predict the prognosis of CC patients, we provided a quantitative approach for clinicians. The approach was a nomogram integrating age, TNM stage and the risk score of the model (Fig. 5b). The chance of survival of a CC patient within 1, 3 and 5 years could be identified by drawing a straight downward line from the overall point axis of the outcome axis. The nomogram’s C-index was 0.804 (95%CI: 0.745–0.863). Relative to the 45-degree line as the ideal curve, the bias-corrected line on the calibration plot was satisfactory (Fig. 5c–e). Overall, these data indicate that the nomogram is a worthwhile model for predicting CC patient survival in both the short and long term and has advantages over the individual diagnostic features.

### A low risk score is linked to a high level of immune infiltration and high cytotoxic potential

**Fig. 6:**
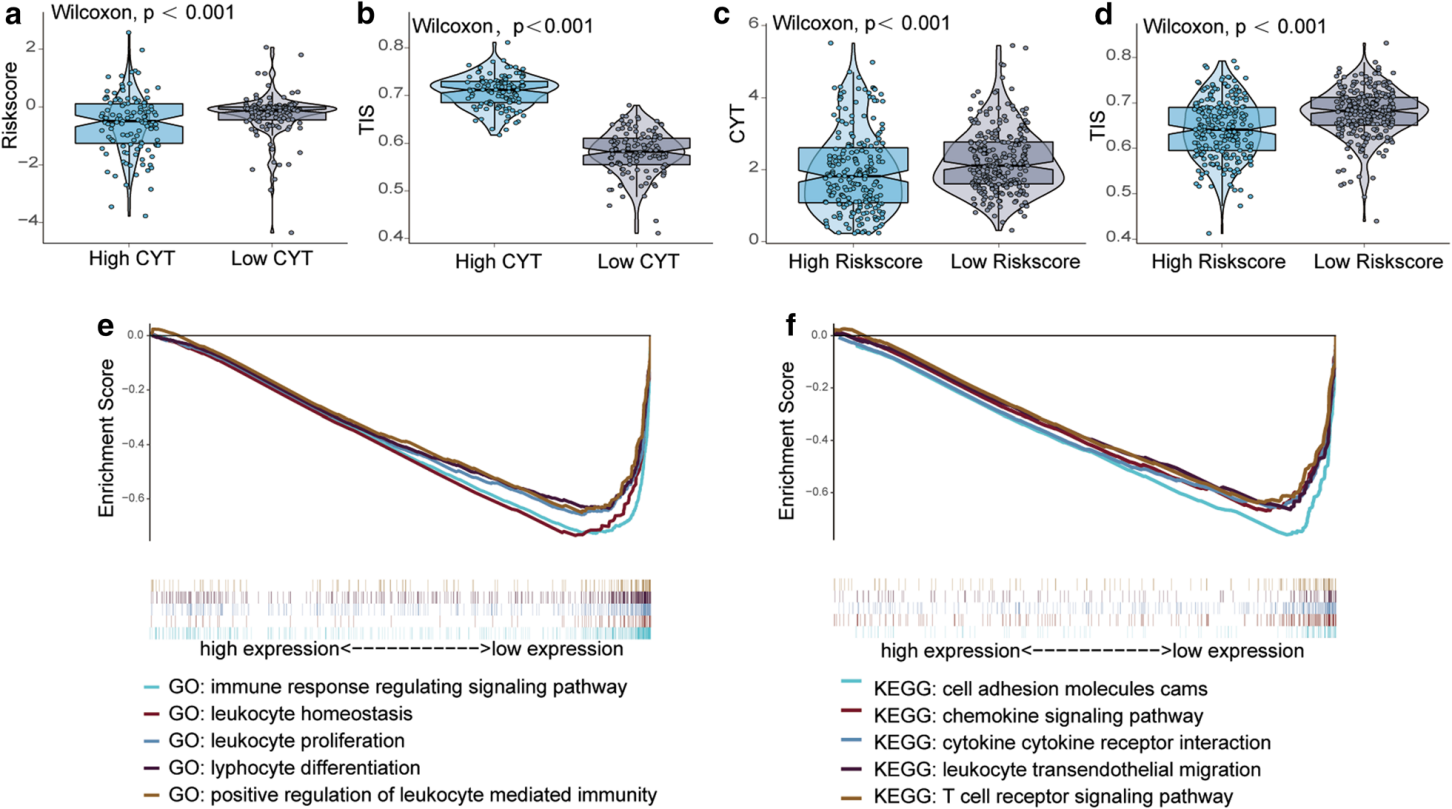
Immune cell infiltration in the low- and high-risk-score groups. **a** Distribution of risk scores in the low- and high-CYT groups. **b** T-cell infiltration score (TIS) in the low- and high-CYT groups. **c** Relative CYT in the low- and high-risk-score groups. **d** Comparison of TIS in the low- and high-risk-score groups. **e** GO analysis of risk scores by GSEA. **f** KEGG analysis of risk scores by GSEA

Samples with high CYT scores had lower risk scores than those with low CYT scores (Fig. 6a). Additionally, low-risk-score samples had high CYT scores (Fig. 6c). The TIS (*P* < 0.001) (Fig. 6b, d) value was increased in the high-CYT-score group and low-risk-score group. GSEA was performed to obtain the GO and KEGG pathways of the model. In the low-risk-score group, the GO analyses showed that leukocyte homeostasis and lymphocyte proliferation were the most significantly enriched biological processes (Fig. 6e), and KEGG pathways T-cell receptor signalling and cytokine-cytokine receptor interaction were significantly downregulated (Fig. 6f). These data indicate that low-risk-score tumours have increased antitumour immune infiltration compared with high-risk-score tumours.

### The two-gene prognostic model can predict the immunotherapeutic benefits in CC

**Fig. 7:**
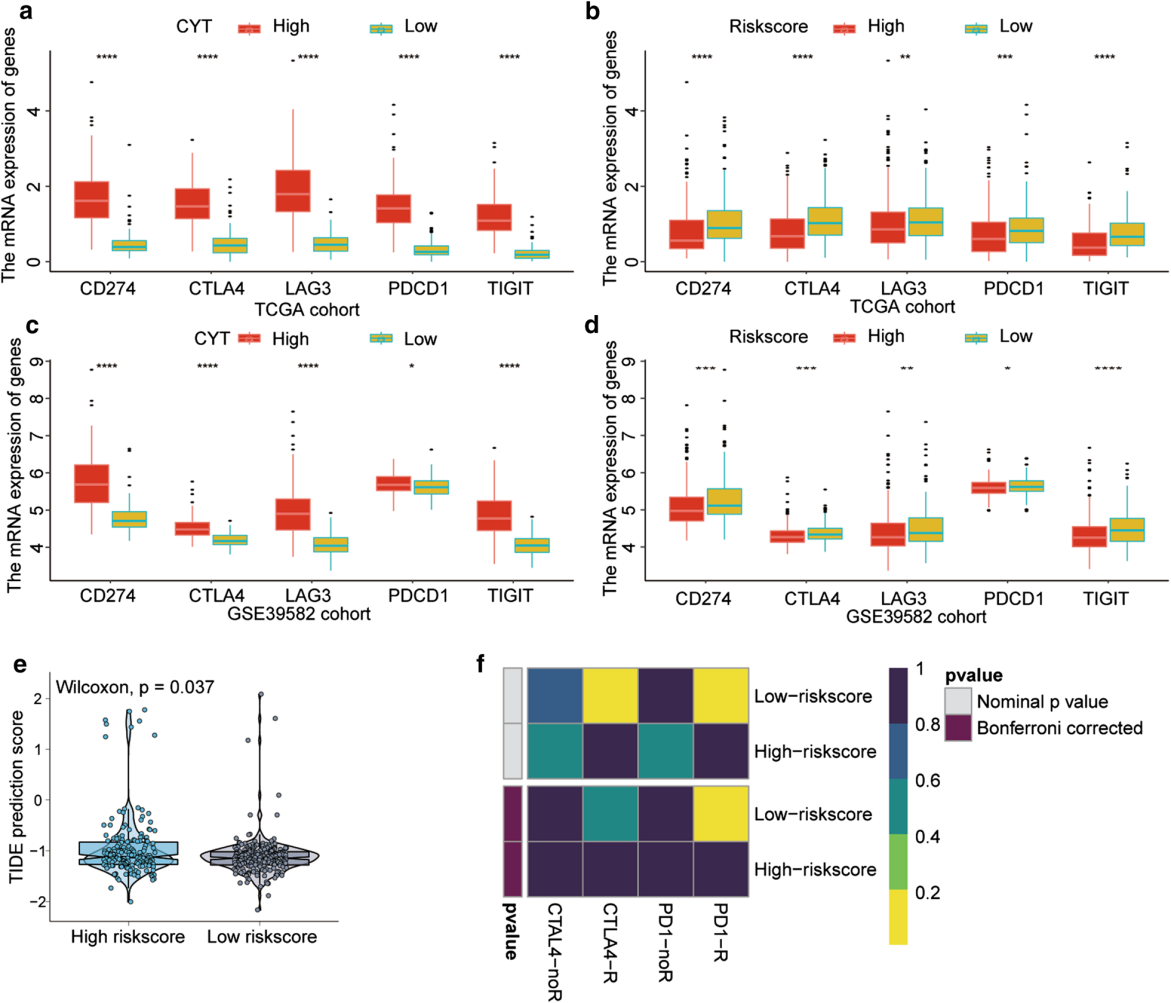
The immune signature predicts immunotherapeutic benefits. **a**, **c** Correlation between CYT with CD274 (PD-L1), CTLA4, LAG3, PDCD1 (PD1) and TIGIT expression in the TCGA cohort and GSE39582. **b**, **d** Correlation between risk score and CD274 (PD-L1), CTLA4, LAG3, PDCD1 (PD1) and TIGIT expression in the TCGA cohort and GSE39582. **e** Comparison of the Tumour Immune Dysfunction and Exclusion (TIDE) prediction scores in the low- and high-risk score groups in the TCGA cohort. **f** Immunotherapeutic responses to anti-CTLA-4 and -PD-1 treatments in high- and low-risk patients

Immune checkpoint blocking therapy for CTLA4 and PD1 has become an effective method for treating various malignant tumours. Therefore, we analysed the relationship of immune checkpoints with the risk-score. Interestingly, we found that the expression levels of CD274 (PDL1), CTLA4, LAG3, PDCD1 (PD1) and TIGIT in patients were increased in the high-CYT score group and low-risk score group (Fig. 7a, b). In the GSE39582 cohort, similar results were observed (Fig. 7c, d). The TIDE algorithm was used to estimate the response to immune checkpoint blockade. The results showed that CC patients with a low-risk-score had a lower-TIDE-score than those with a high-risk-score (*P* = 0.037; Fig. 7e), indicating that low-risk-score CC patients have more promise in response to anti-immune checkpoint therapy. In addition, using subclass mapping, we found that the expression profile of CC patients in the low-risk group was correlated with that in the PDL1 response group (*P* = 0.014; Fig. 7f). Therefore, these results suggest that the model can predict the benefit of CC immunotherapy.

## Discussion

Currently, many researchers are searching biomarkers to predict the immune response. In 2015, the CYT score was presented by Michael S. Rooney et al. who quantified the cytolytic activity of the local immune infiltrate and proposed and demonstrated that CYT is associated with counter-regulatory immune responses [9]. In addition, Apostolos Zaravinos et al. found that the cytolytic activity is correlated with distinct mutational events in CRC, and cytokine and immune checkpoint expression patterns differ in colorectal tumours with high versus low cytolytic activity [10]. In this study, CYT-related genes were identified for the first time, providing new biomarkers and therapeutic targets for the prognostic analysis of CC. The genes screened by CYT are specifically enriched in the GO biological processes and KEGG pathway associated with the immune response. Therefore, we developed and validated a prognostic model based on two genes (HOXC8 and MS4A2). The model identified high-risk CC patients with a poor prognosis.

As a member of the homeobox gene group [28], HOXC8 translates homeodomain-containing transcription elements that are vital for body patterning, development, and differentiation in vertebrates and invertebrates [29]. This transcription element is adept at stimulating oncogenes among several malignancies and is linked to the modulation of many proteins associated with cancers [30], such as breast cancer [31] and prostate cancer [32]. In patients with hepatocellular carcinoma, HOXC8 expression is related to poor survival and recurrence [33]. In our study, HOXC8 expression was upregulated in CC compared with normal controls.

MS4A2 is a marker of mast cells [34]. Previous studies have reported that MS4A2 is expressed on mast cells and basophils at a high density [35]. In the tumour microenvironment, mast cells and basophils can produce various immune mediators, which can interact with immune and non-immune components to help regulate the immune response [36]. Several studies have reported that a high density of mast cells in tumours is related to favourable survival in CC patients [37, 38]. Dalam Ly et al. found that MS4A2-positive cells are localised in the tumour stroma and co-stain with the mast cell-specific protease [39]. Lei Sun et al. found that MS4A2 is a favourable prognostic factor in gastric cancer [40]. Our study found that the expression of MS4A2 is downregulated in CC compared with that in normal tissues or adjacent tissues through database analyses and PCR and IHC experiments.

We found that high expression levels of HOXC8 and low expression levels of MS4A2 are related to poor prognoses in CC patients. In addition, the risk score is positively correlated with the TNM stage, T stage and N stage. Moreover, we found that the model is considered an independent factor for prognostics. Then, a nomogram that combines the risk score of the model with other clinical features (age, T stage, N stage, M stage and the risk score) was proposed. The nomogram was associated with the survival of individual patients, providing patients with a respective scoring system. Hence, the nomogram is a viable instrument for use by clinicians moving forward [22].

In antitumour immunity, the T-cell immune response is the central event, and CD8 + T cells (cytotoxic T cells) are the most important antitumour cells [41]. Cytotoxic T cells, memory T cells and Th1 cells related to a favourable survival outcome [24]. Therefore, patients with high TIS and CYT values may present stronger antitumour effects than other patients. Combined with the GSEA results, these data indicate that the low-risk-score group had increased levels of antitumour immunity compared with the high-risk-score group.

According to the hypothesis of cancer immunoediting, to alleviate the antitumour immune response during the development of cancer in the immunocompetent host, tumours prefer tissues with lower immunogenicity during the growth process [42]. Therefore, clinically meaningful cancers have several mechanisms involved in immunosuppression, such as enhancing different cells to participate in immunosuppression and enhancing various molecules to participate in immunosuppression, including CTLA4, PD1, PDL1, LAG3 and other immune checkpoints. Although CTLA4 and PD1-PDL1-targeted cancer immunotherapy has had a profound impact on the treatment of CC, treatment is ineffective in a large proportion of patients [43]. Studies have found that the increase in PD1, PDL1, and lymphocyte infiltration in tumour cells can increase the response rate to anti-PD1/PDL1 therapy. If PD1 is overexpressed without cytotoxic T-cell infiltration, immunotherapy is ineffective [6]. In our analysis, the expression of PD1, PDL1, LAG3, CTLA4 and TIGIT was increased in the low-risk-score group compared with that in the high-risk score group. The TIDE algorithm and subclass mapping were used to estimate the response to immune checkpoint blockade. The foundings suggest that the model can predict the benefit of CC immunotherapy. Thus, the poor prognosis of high-risk patients with CC may be due to the low immunosuppression and immune response in the tumour microenvironment, which promotes tumour growth, invasion and metastasis. Importantly, because of these differences, low-risk patients with CC may predict the benefit of CC immunotherapy.

## Conclusion

Our study developed and validated a two-gene prognostic model, which may be a predictive factor for the response to immunotherapy. However, the study is limited because it was retrospective, and further experimental studies are needed to confirm these results and reveal their underlying mechanisms. In summary, this study is the first to identify a two-gene prognostic model associated with CYT that independently predicts survival in CC patients. In addition, it can guide clinical treatment by predicting the response to anti-immune checkpoint treatment to some extent.

## Supplementary Information

**Additional file 1**: **Table S1**: Clinical features of CC patients in training set and testing set.

**Additional file 2**: **Table S2**: Primer sequences for RT-PCR.

**Additional file 3**: **Table S3**: Clinicopathological parameters of CC patients in the CC cohort for IHC.

**Additional file 4**: **Table S4**: The univariate Cox HR regression result of selected genes.

**Additional file 5**: **Table S5**: The specific statistical parameters of HOXC8 and MS4A2 in each group.

**Additional file 6**: **Figure S1**: Flow chart of the study. Five public colon cancer-related datasets were chosen for this study. First, we obtained CYT-related DEGs. Next, a two-gene prognostic model associated with CYT was established and validated. Finally, we verified the correlation between the model and T-cell infiltration. **Figure S2**: a-b LASSO Cox analysis identified the two genes most correlated with overall survival (OS). c-d Effect of HOXC8 and MS4A2 expression on the OS of CC patients in the whole TCGA cohort. **Figure S3**: Kaplan-Meier survival, risk score and time-dependent receiver operating characteristic (ROC) curves of the model for the testing cohort a-c and whole cohort d-f in CC. a, d Overall survival (OS) was significantly higher in the low-risk-score group than in the high-risk-score group. b, e The relationship between the risk score (upper panel) and expression of two prognostic immune genes (lower panel) is shown. c, f Time-dependent ROC curve analysis. Relationship between the expression of the risk score and g the TNM stage, h T stage, i M stage and j N stage in the IHC cohort.

## Data Availability

The obtained data were used according to the TCGA (https://portal.gdc.cancer.gov/repository), GTEx (http://commonfund.nih.gov/GTEx) and GEO (https://www.ncbi.nlm.nih.gov/geo/) data access policies. The mutation data, mRNA profile data and clinical feature data for CC are publicly obtainable and open access. All analyses were carried out based on pertinent guidelines and regulations.

## Abbreviations

CYT: Cytotoxic activity scores
CC: Colon cancer
COAD: Colon adenocarcinoma
TCGA: The Cancer Genome Atlas
TIDE: Tumour Immune Dysfunction and Exclusion
GEO: Gene expression omnibus
ssGSEA: Single-sample gene set enrichment analysis
TIS: The T cell infiltration score
TILs: Tumour-infiltrating lymphocytes
CYT: Cytotoxic activity scores
FPKM: Fragments per kilobase of transcript per million mapped reads;
AJCC: American Joint Committee on Cancer
GSEA: Gene set enrichment analysis
PD1: Programmed death 1
DEGs: Differentially expressed genes
log2FC: Log2|fold change|
FDR: False discovery rate
GO: Gene ontology
KEGG: Kyoto Encyclopedia of Genes and Genomes
HOXC8: Homeobox C8
MS4A2: Membrane Spanning 4-Domains A2
TNM: Tumor‑node‑metastasis
LASSO: Least absolute shrinkage and selection operator
OS: Overall survival
ROC: Receiver operating characteristic
C-index: Concordance index;
RT-qPCR: Reverse transcription-quantitative polymerase
IHC: Immunohistochemistry
PBS: Phosphate buffered saline
DAB: Diaminobenzidine
