# Supplementary material for: Identification of a two-gene prognostic model associated with cytolytic activity for colon cancer
Source: Cancer Cell Int. 2021 Feb 8;21:95. doi: 10.1186/s12935-021-01782-6 (PMC7869500; doi:10.1186/s12935-021-01782-6)
Supplement: Supplementary file 1 — Additional file 1: Table S1: Clinical features of CC patients in training set and testing set. [file 12935_2021_1782_MOESM1_ESM.docx]

**Table S1** Clinical features of CC patients in training set and testing set

| **Clinical features** | **Testing set**  **(n=181)** | **Training set**  **(n=181)** | ***χ2*** | ***P*.value** |
| --- | --- | --- | --- | --- |
| Age , y |  |  |  |  |
| ≥ 65 | 134 | 129 | 0.348 | 0.555 |
| < 65 | 47 | 52 |  |  |
| Gender |  |  |  |  |
| MALE | 105 | 90 | 2.501 | 0.114 |
| FEMALE | 76 | 91 |  |  |
| Pathologic-stage |  |  |  |  |
| I | 30 | 35 | 1.74 | 0.628 |
| II | 69 | 76 |  |  |
| III | 51 | 45 |  |  |
| IV | 31 | 25 |  |  |
| TNM-T |  |  |  |  |
| T1 | 4 | 4 | 0.687 | 0.876 |
| T2 | 29 | 35 |  |  |
| T3 | 127 | 122 |  |  |
| T4 | 21 | 20 |  |  |
| TNM-M |  |  |  |  |
| M0 | 150 | 156 | 0.761 | 0.383 |
| M1 | 31 | 25 |  |  |
| TNM-N |  |  |  |  |
| N0 | 103 | 115 | 1.918 | 0.382 |
| N1 | 44 | 40 |  |  |
| N2 | 34 | 26 |  |  |
| Total | 181 | 181 |  |  |

Abbreviations:T tumor, N node, M metastasis
