# Supplementary material for: Identification of a two-gene prognostic model associated with cytolytic activity for colon cancer
Source: Cancer Cell Int. 2021 Feb 8;21:95. doi: 10.1186/s12935-021-01782-6 (PMC7869500; doi:10.1186/s12935-021-01782-6)
Supplement: Supplementary file 2 — Additional file 2: Table S2: Primer sequences for RT-PCR. [file 12935_2021_1782_MOESM2_ESM.docx]

**Table S2** Primer sequences for RT-PCR

| **Gene symbol** | **Primer sequence** |
| --- | --- |
| **HOXC8** | Forward: 5′-TTCTTCCACCACGGCACCTC-3′  Reverse: 5′-GCCTCTTGCTGAGCCCCATA-3′ |
| **MS4A2** | Forward: 5′-TCTGGGACTTGGTAGTGCTGTG-3′  Reverse: 5′-CCTGGGTCTTCCAACTCACTG-3′ |
| **β-actin** | Forward: 5′-CCAGAAACTACCTTCAACTCC-3′  Reverse: 5′-GTGATCTCCTTGTGCATCCTGT-3′ |

**the NCBI Reference Sequence：**

HOXC8: ATGAGCTCCTACTTCGTCAACCCCCTGTTCTCCAAATACAAAGCCGGCGAGTCCCTGGAACCGGCCTATT

ACGACTGCCGGTTCCCTCAGAGCGTGGGCAGGAGCCATGCGCTGGTGTACGGGCCCGGCGGCTCGGCGCC

CGGCTTCCAGCACGCTTCGCACCACGTTCAAGACTTCTTCCACCACGGCACCTCCGGCATCTCCAACTCA

GGCTACCAGCAGAACCCGTGCTCGCTTAGCTGCCACGGAGACGCCTCCAAATTCTATGGCTACGAGGCGC

TCCCCAGACAGTCCCTTTATGGGGCTCAGCAAGAGGCGAGCGTGGTGCAATATCCCGACTGTAAATCCTC

CGCCAACACTAACAGTAGCGAAGGACAAGGCCACTTAAATCAAAACTCGTCTCCCAGCCTCATGTTTCCA

TGGATGAGACCCCACGCTCCGGGGAGGCGCAGTGGACGGCAAACTTACAGCCGGTATCAGACCTTGGAAC

TAGAAAAGGAGTTTCTCTTTAATCCTTATTTGACACGAAAACGTCGGATTGAAGTCTCTCATGCCCTGGG

ACTGACCGAGAGACAAGTGAAGATCTGGTTCCAGAACCGAAGGATGAAGTGGAAAAAGGAGAACAACAAG

GATAAACTGCCGGGAGCCCGAGATGAGGAGAAGGTGGAGGAAGAAGGAAATGAGGAAGAGGAGAAAGAAG

AGGAGGAAAAGGAAGAAAACAAGGACTAA

MS4A2:

ATGAGCTCGGTTAATGAAAAAATGGACACAGAAAGTAATAGGAGAGCAAATCTTGCTCTCCCACAGGAGC

CTTCCAGTGTGCCTGCATTTGAAGTCTTGGAAATATCTCCCCAGGAAGTATCTTCAGGCAGACTATTGAA

GTCGGCCTCATCCCCACCACTGCATACATGGCTGACAGTTTTGAAAAAAGAGCAGGAGTTCCTGGGGGTA

ACACAAATTCTGACTGCTATGATATGCCTTTGTTTTGGAACAGTTGTCTGCTCTGTACTTGATATTTCAC

ACATTGAGGGAGACATTTTTTCATCATTTAAAGCAGGTTATCCATTCTGGGGAGCCATATTTTTTTCTAT

TTCTGGAATGTTGTCAATTATATCTGAAAGGAGAAATGCAACATATCTGGTGAGAGGAAGCCTGGGAGCA

AACACTGCCAGCAGCATAGCTGGGGGAACGGGAATTACCATCCTGATCATCAACCTGAAGAAGAGCTTGG

CCTATATCCACATCCACAGTTGCCAGAAATTTTTTGAGACCAAGTGCTTTATGGCTTCCTTTTCCACTGA

AATTGTAGTGATGATGCTGTTTCTCACCATTCTGGGACTTGGTAGTGCTGTGTCACTCACAATCTGTGGA

GCTGGGGAAGAACTCAAAGGAAACAAGGTTCCAGAGGATCGTGTTTATGAAGAATTAAACATATATTCAG

CTACTTACAGTGAGTTGGAAGACCCAGGGGAAATGTCTCCTCCCATTGATTTATAA

β-actin:

ATGGATGATGATATCGCCGCGCTCGTCGTCGACAACGGCTCCGGCATGTGCAAGGCCGGCTTCGCGGGCG

ACGATGCCCCCCGGGCCGTCTTCCCCTCCATCGTGGGGCGCCCCAGGCACCAGGGCGTGATGGTGGGCAT

GGGTCAGAAGGATTCCTATGTGGGCGACGAGGCCCAGAGCAAGAGAGGCATCCTCACCCTGAAGTACCCC

ATCGAGCACGGCATCGTCACCAACTGGGACGACATGGAGAAAATCTGGCACCACACCTTCTACAATGAGC

TGCGTGTGGCTCCCGAGGAGCACCCCGTGCTGCTGACCGAGGCCCCCCTGAACCCCAAGGCCAACCGCGA

GAAGATGACCCAGATCATGTTTGAGACCTTCAACACCCCAGCCATGTACGTTGCTATCCAGGCTGTGCTA

TCCCTGTACGCCTCTGGCCGTACCACTGGCATCGTGATGGACTCCGGTGACGGGGTCACCCACACTGTGC

CCATCTACGAGGGGTATGCCCTCCCCCATGCCATCCTGCGTCTGGACCTGGCTGGCCGGGACCTGACTGA

CTACCTCATGAAGATCCTCACCGAGCGCGGCTACAGCTTCACCACCACGGCCGAGCGGGAAATCGTGCGT

GACATTAAGGAGAAGCTGTGCTACGTCGCCCTGGACTTCGAGCAAGAGATGGCCACGGCTGCTTCCAGCT

CCTCCCTGGAGAAGAGCTACGAGCTGCCTGACGGCCAGGTCATCACCATTGGCAATGAGCGGTTCCGCTG

CCCTGAGGCACTCTTCCAGCCTTCCTTCCTGGGCATGGAGTCCTGTGGCATCCACGAAACTACCTTCAAC

TCCATCATGAAGTGTGACGTGGACATCCGCAAAGACCTGTACGCCAACACAGTGCTGTCTGGCGGCACCA

CCATGTACCCTGGCATTGCCGACAGGATGCAGAAGGAGATCACTGCCCTGGCACCCAGCACAATGAAGAT

CAAGATCATTGCTCCTCCTGAGCGCAAGTACTCCGTGTGGATCGGCGGCTCCATCCTGGCCTCGCTGTCC

ACCTTCCAGCAGATGTGGATCAGCAAGCAGGAGTATGACGAGTCCGGCCCCTCCATCGTCCACCGCAAAT

GCTTCTAG

**Real-time PCR parameters**

Parameters of real-time PCR were: initial denaturation and enzyme activation at 95 °C for 30 s, denaturation at 95 °C for 5 s, annealing at 60 °C for 30 s, 40 cycles.
