# Supplementary material for: Identification of a two-gene prognostic model associated with cytolytic activity for colon cancer
Source: Cancer Cell Int. 2021 Feb 8;21:95. doi: 10.1186/s12935-021-01782-6 (PMC7869500; doi:10.1186/s12935-021-01782-6)
Supplement: Supplementary file 3 — Additional file 3: Table S3: Clinicopathological parameters of CC patients in the CC cohort for IHC. [file 12935_2021_1782_MOESM3_ESM.docx]

**Table S3. Clinicopathological parameters of CC patients in the CC cohort for IHC.**

| **Variable** | **Patients in the CC cohort for IHC** | |
| --- | --- | --- |
|  | **n** | **%** |
| **Age(y)** |  |  |
| <65 | 20 | 50 |
| ≥65 | 20 | 50 |
| **Gender** |  |  |
| Male | 20 | 50 |
| Female | 20 | 50 |
| **Clinical stage** |  |  |
| Ⅰ | 5 | 12.5 |
| Ⅱ | 13 | 32.5 |
| Ⅲ | 12 | 30 |
| Ⅳ | 10 | 25 |
| **T classification** |  |  |
| T1 | 4 | 10 |
| T2 | 2 | 5 |
| T3 | 16 | 40 |
| T4 | 18 | 45 |
| **N classification** |  |  |
| N0 | 22 | 55 |
| N1 | 12 | 30 |
| N2 | 6 | 15 |
| **Metastasis** |  |  |
| No | 30 | 75 |
| Yes | 10 | 25 |
