# Supplementary material for: Identification of a two-gene prognostic model associated with cytolytic activity for colon cancer
Source: Cancer Cell Int. 2021 Feb 8;21:95. doi: 10.1186/s12935-021-01782-6 (PMC7869500; doi:10.1186/s12935-021-01782-6)
Supplement: Supplementary file 4 — Additional file 4: Table S4: The univariate Cox HR regression result of selected genes. [file 12935_2021_1782_MOESM4_ESM.docx]

| Table 4. The univariate Cox HR regression result of selected genes | | | | | |
| --- | --- | --- | --- | --- | --- |
| Genes | HR | HR.95L | HR.95H | pvalue | Log_2_FC |
| ZNF114 | 2.135035 | 1.069924 | 4.260463 | 0.031421 | 1.668323 |
| VIM-AS1 | 1.723392 | 1.036667 | 2.865026 | 0.035832 | -1.02331 |
| TNIP3 | 0.409485 | 0.171015 | 0.980484 | 0.045048 | 1.113144 |
| ROBO3 | 2.227514 | 1.229791 | 4.034683 | 0.008232 | -1.22903 |
| POMC | 2.301208 | 1.158701 | 4.570251 | 0.017278 | -2.38834 |
| PDE1B | 2.39516 | 1.386987 | 4.136155 | 0.001727 | -1.93465 |
| PCOLCE2 | 2.069786 | 1.378701 | 3.107284 | 0.00045 | -1.87256 |
| NMRAL2P | 1.733804 | 1.155475 | 2.601592 | 0.007862 | -1.11614 |
| MS4A2 | 0.341083 | 0.124768 | 0.932431 | 0.036055 | -1.22692 |
| LINC01614 | 1.368653 | 1.025565 | 1.826515 | 0.033055 | 4.155508 |
| LINC00941 | 1.390693 | 1.048542 | 1.844493 | 0.022083 | 2.81662 |
| KRT16 | 1.207187 | 1.011437 | 1.44082 | 0.036983 | 1.937143 |
| HOXC9 | 2.011373 | 1.259465 | 3.212174 | 0.003436 | 1.308123 |
| HOXC8 | 2.766263 | 1.650512 | 4.636265 | 0.000113 | 1.045953 |
| HDC | 0.323237 | 0.118174 | 0.88414 | 0.02782 | -1.58808 |
| HAS1 | 1.660053 | 1.088586 | 2.531521 | 0.018562 | -1.42972 |
| FCRL2 | 2.578777 | 1.231524 | 5.399886 | 0.011997 | -1.22409 |
| FCER2 | 1.839735 | 1.138264 | 2.973496 | 0.012823 | -1.18634 |
| FAM110D | 1.875965 | 1.057419 | 3.328146 | 0.031489 | -1.57659 |
| FABP4 | 1.188779 | 1.002441 | 1.409754 | 0.046812 | -1.85592 |
| EBF3 | 3.356397 | 1.564816 | 7.199186 | 0.001871 | -1.61203 |
| DPYSL4 | 1.974354 | 1.039556 | 3.749748 | 0.037663 | -1.35742 |
| CCL22 | 0.700369 | 0.492852 | 0.995263 | 0.046984 | 1.745358 |
| CADM3 | 1.737177 | 1.01232 | 2.981055 | 0.045026 | -3.04387 |
| B3GALT5-AS1 | 0.292331 | 0.085965 | 0.994099 | 0.048902 | -1.30658 |
| AC243967.1 | 0.281626 | 0.087357 | 0.907923 | 0.033863 | 2.465316 |
