# Supplementary material for: Identification of a two-gene prognostic model associated with cytolytic activity for colon cancer
Source: Cancer Cell Int. 2021 Feb 8;21:95. doi: 10.1186/s12935-021-01782-6 (PMC7869500; doi:10.1186/s12935-021-01782-6)
Supplement: Supplementary file 5 — Additional file 5: Table S5: The specific statistical parameters of HOXC8 and MS4A2 in each group. [file 12935_2021_1782_MOESM5_ESM.docx]

**Table S5** The specific statistical parameters of HOXC8 and MS4A2 in each group

| Genes | Groups | Normal | | Tumour | | Log_2_FC | P |
| --- | --- | --- | --- | --- | --- | --- | --- |
|  |  | Mean | SD | Mean | SD |  |  |
| HOXC8 | GTEx+TCGA cohort | 0.113 | 0.287 | 0.225 | 0.321 | 1.003 | P＜0.001 |
|  | Groups for qPCR | 0.097 | 0.148 | 0.490 | 0.519 | 2.342 | P＜0.001 |
|  | Groups for IHC | 2.113 | 0.749 | 2.656 | 0.632 | 0.330 | P = 0.001 |
| MS4A2 | GTEx+TCGA cohort | 1.113 | 0.784 | 0.475 | 0.480 | 1.227 | P＜0.001 |
|  | Groups for qPCR | 0.094 | 0.136 | 0.003 | 0.008 | 4.973 | P＜0.001 |
|  | Groups for IHC | 2.175 | 0.359 | 1.856 | 0.650 | 0.229 | P = 0.003 |
