# Supplementary material for: Identification of a two-gene prognostic model associated with cytolytic activity for colon cancer
Source: Cancer Cell Int. 2021 Feb 8;21:95. doi: 10.1186/s12935-021-01782-6 (PMC7869500; doi:10.1186/s12935-021-01782-6)

**Supplementary Figure legends**

**Figure S1** Flow chart of the study. Five public colon cancer-related datasets were chosen for this study. First, we obtained CYT-related DEGs. Next, a two-gene prognostic model associated with CYT was established and validated. Finally, we verified the correlation between the model and T-cell infiltration.

**
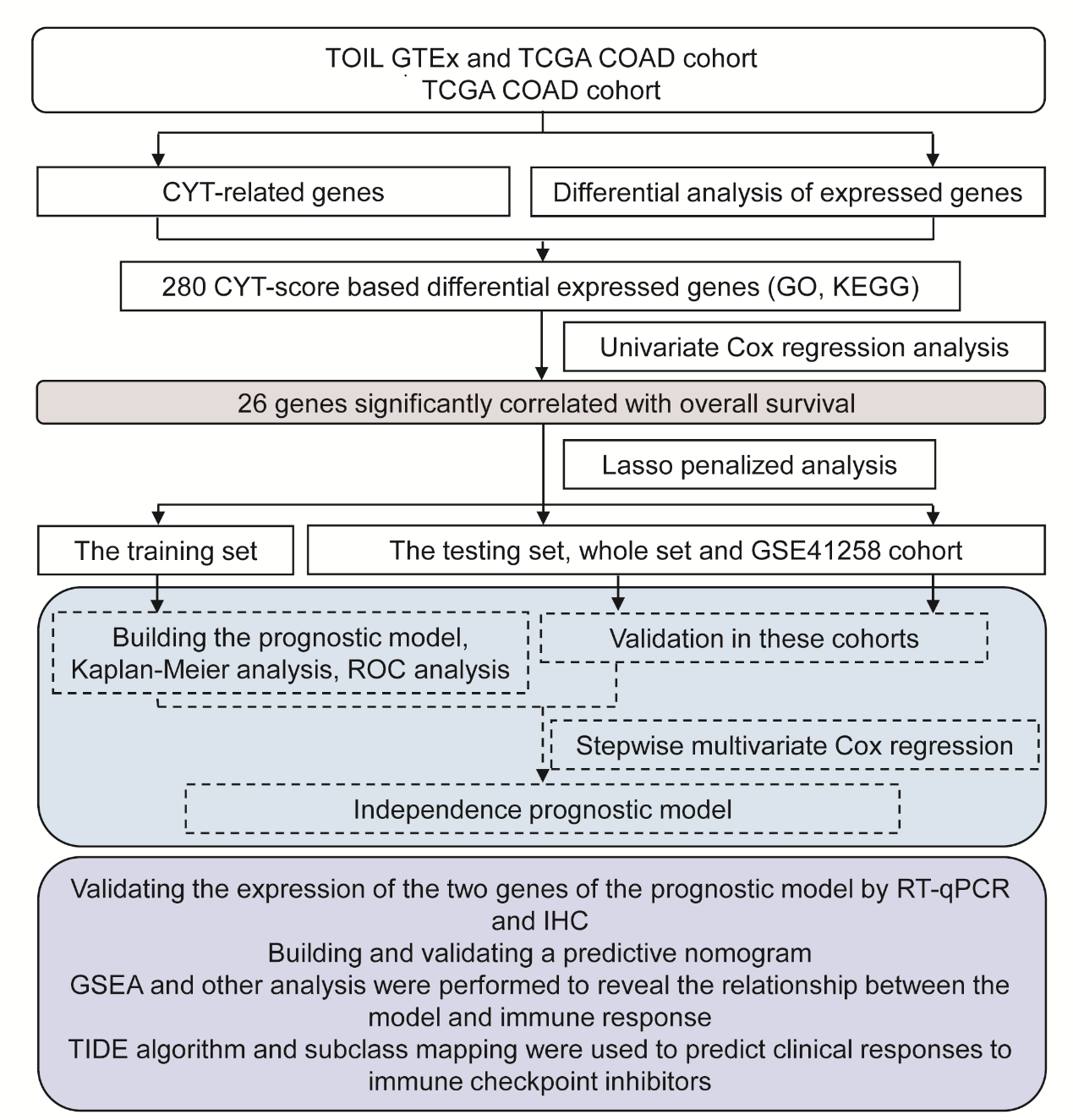
**

**Figure S2** a-b LASSO Cox analysis identified the two genes most correlated with overall survival (OS). **c-d** Effect of HOXC8 and MS4A2 expression on the OS of CC patients in the whole TCGA cohort.


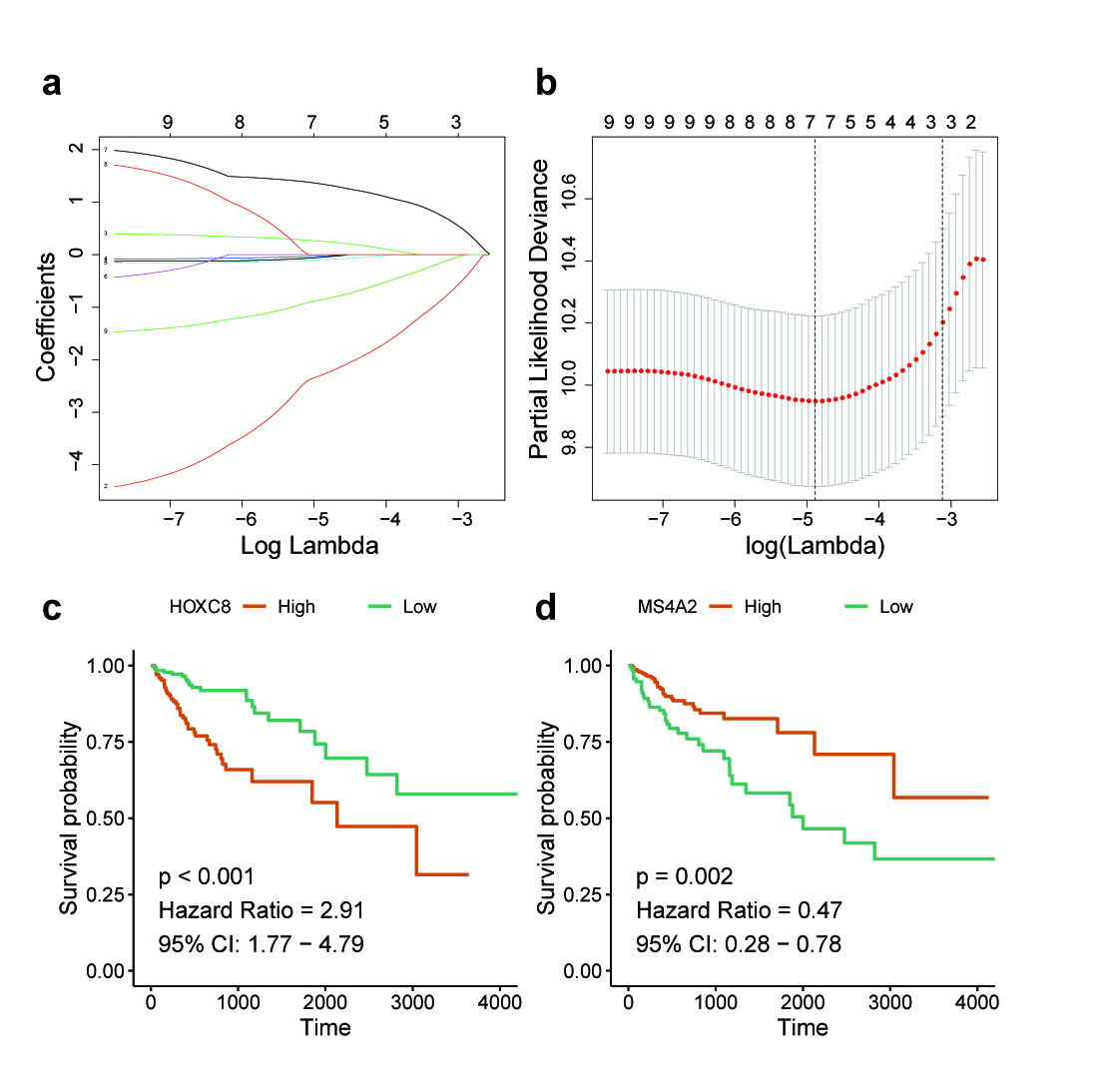


**Figure S3** Kaplan-Meier survival, risk score and time-dependent receiver operating characteristic (ROC) curves of the model for the testing cohort **a-c** and whole cohort **d-f** in CC. **a, d** Overall survival (OS) was significantly higher in the low-risk-score group than in the high-risk-score group. **b, e** The relationship between the risk score (upper panel) and expression of two prognostic immune genes (lower panel) is shown. **c, f** Time-dependent ROC curve analysis. Relationship between the expression of the risk score and **g** the TNM stage, **h** T stage, **i** M stage and **j** N stage in the IHC cohort.


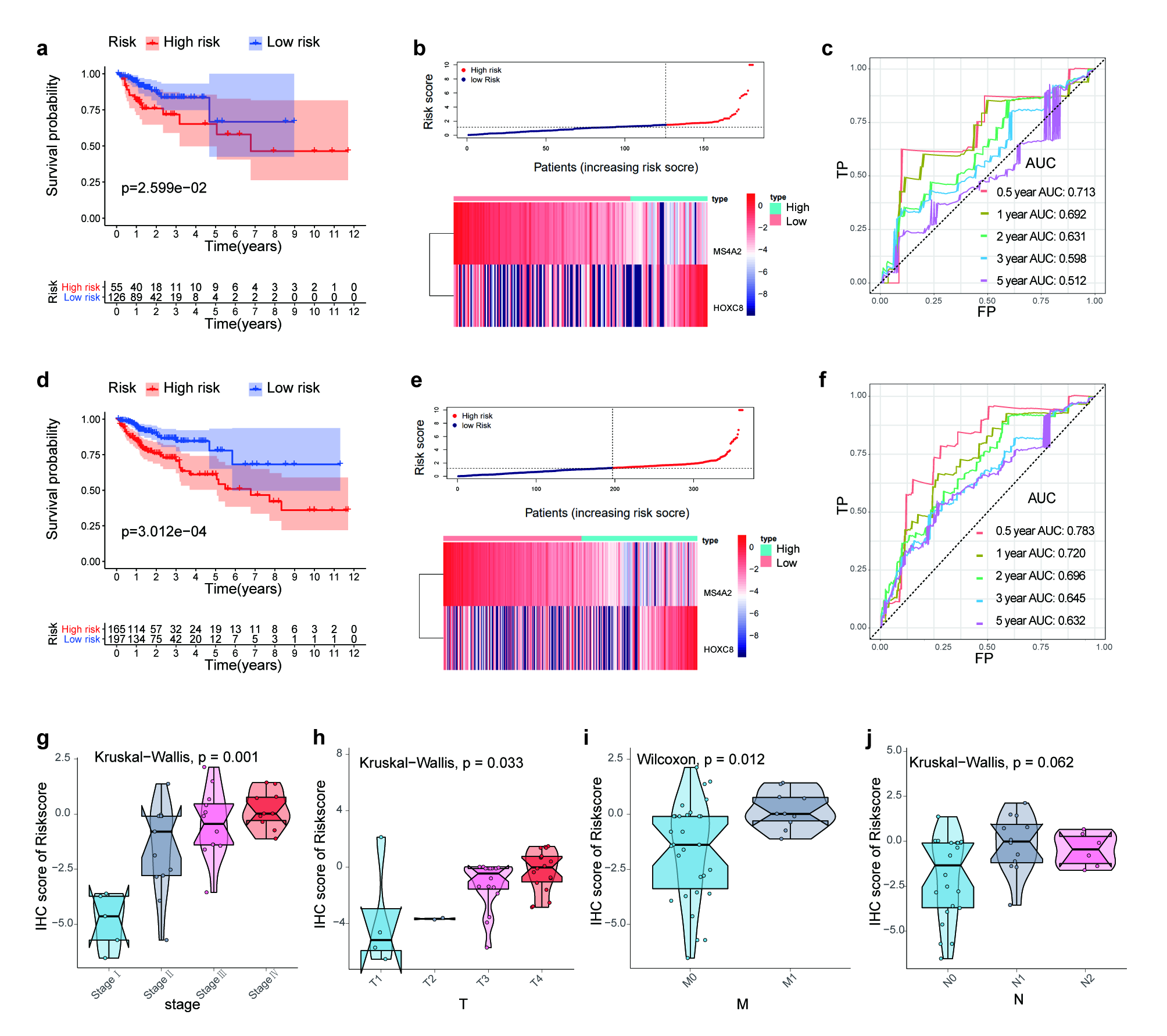

Supplement: Supplementary file 6 — Additional file 6: Figure S1: Flow chart of the study. Five public colon cancer-related datasets were chosen for this study. First, we obtained CYT-related DEGs. Next, a two-gene prognostic model associated with CYT was established and validated. Finally, we verified the correlation between the model and T-cell infiltration. Figure S2: a-b LASSO Cox analysis identified the two genes most correlated with overall survival (OS). c-d Effect of HOXC8 and MS4A2 expression on the OS of CC patients in the whole TCGA cohort. Figure S3: Kaplan-Meier survival, risk score and time-dependent receiver operating characteristic (ROC) curves of the model for the testing cohort a-c and whole cohort d-f in CC. a, d Overall survival (OS) was significantly higher in the low-risk-score group than in the high-risk-score group. b, e The relationship between the risk score (upper panel) and expression of two prognostic immune genes (lower panel) is shown. c, f Time-dependent ROC curve analysis. Relationship between the expression of the risk score and g the TNM stage, h T stage, i M stage and j N stage in the IHC cohort. [file 12935_2021_1782_MOESM6_ESM.docx]
